# Willingness and determinants of elder care modes among elderly individuals: insights from underdeveloped regions in Western China

**DOI:** 10.7189/jogh.15.04031

**Published:** 2025-01-24

**Authors:** Yunhua Wang, Guorong Chai, Jiancheng Wang

**Affiliations:** 1 School of Management, Lanzhou University, Gansu, China; 2 Gansu Health Vocational College, Gansu, China

## Abstract

**Background:**

The aim of the present study was to investigate the willingness of elderly individuals regarding their choice of elderly care modes in underdeveloped regions of Western China and to identify the key factors influencing the willingness.

**Methods:**

We distributed a total of 20 000 questionnaires using the multistage stratified cluster random sampling method, and successfully collected 19 460 of them. After conducting quality checks, we deemed 19 040 questionnaires valid for analysis. The survey covered seven cities, 24 counties, and 255 villages in Gansu Province. We conducted statistical analyses, including univariate χ^2^ test, Kruskal-Wallis test and multivariate logistic regression, to assess the willingness of elderly individuals regarding care modes and the factors influencing these choices.

**Results:**

Among the 19 040 valid responses, 88.55% preferred home care, 5.01% opted for community care, 5.01% chose institutional care, 1.08% preferred mutual care, and 0.36% selected smart care. Elderly individuals who have a lower level of education, residence within a 15-minute walking distance to the nearest health care facility, and believe that elder care should rely on themselves or their children, etc. exhibit a higher willingness for choosing the home care mode (*P* < 0.05). Elderly individuals who believe that elderly care should be reliant on their children, and possess a greater knowledge for the combination of medical and elderly care, etc. exhibit a stronger willingness for choosing the community care (*P* < 0.05). Elderly individuals with lower educational level and lack endowment insurance, etc. exhibit a higher willingness for choosing the mutual care (*P* < 0.05). Elderly individuals who are not afflicted with chronic illnesses and reside within a 15-minute distance from the nearest medical centre exhibit a higher willingness for choosing the smart care mode (*P* < 0.05).

**Conclusions:**

In underdeveloped regions in China, home care continues to be the predominant choice among elderly individuals. However, the growing demand for diverse elderly care modes warrants attention. Multiple factors influence the willingness of elderly individuals when selecting care modes. This study offers valuable insights for policymakers, enabling government departments to implement targeted strategies and interventions to meet the diverse service needs of the elderly population effectively.

The aging of China's population is accelerating rapidly. According to the latest data from the National Bureau of Statistics, by the end of 2023, China's population aged 60 years and above had reached 296.97 million, representing 21.1% of the total population. This demographic milestone signifies that China has entered a moderately aging society [1,2]. Concurrently, the declining number of children and the shrinking size of families have made it increasingly challenging for families to bear the full burden of elder care [3]. Further, the aging population trend is intensifying, accompanied by an increase in the life expectancy of the elderly, further exacerbating the demand for comprehensive elder care solutions. In 2020, the number of elderly people aged 80 and above in China was 35.8 million, disabled and semi-disabled elderly in China accounted for about 16.2% of the total elderly population [4]. Compared with their non-disabled counterparts, the disabled elderly are generally in poor health and have many unmet care needs. Moreover, China's economic development has not kept pace with the aging population, resulting in a situation where the nation is 'growing old before growing rich' [5]. These challenges underscore the urgent need to address elder care as a critical societal issue in China.

At present, elder care in China is primarily categorised into three main modes: home care, community care, and institutional care [6–8]. In recent years, innovative modes such as mutual care and smart care have emerged, evolving from these traditional care models to meet the changing needs of the aging population [9,10]. The establishment of the elderly care system is closely related to the willingness of elderly individuals in selecting care modes. The factors influencing the willingness are multifaceted. A review of existing research indicates that studies in developed countries typically focus on personal and structural factors, such as the availability of social public service facilities, affordable housing options for the elderly, and economic support mechanisms for older adults. These factors play a significant role in shaping the care choices of elderly populations in those regions [11–14]. In China, research on the factors that influence the elder care mode is mainly classified based on social demography, which can be roughly divided into four aspects: personal status, family status, socioeconomic status, and health status [6,15–17]. Specifically, research indicates that various demographic and socioeconomic factors influence elderly individuals' preferences for care modes. Males, those with lower monthly incomes, poorer self-assessed health, and a larger number of children are more likely to prefer home-based care [6,11,12,18]. In contrast, elderly individuals with higher educational attainment and those living alone are more inclined toward community-based care [18]. Moreover, individuals with poorer self-assessed health, greater financial resources, enrolment in medical insurance, and unmarried status demonstrate a stronger preference for institutional care [6,19]. Additionally, other studies highlight that better health status and higher monthly incomes are associated with a greater preference for home-based care [12,16].

Research on elderly individuals' preferences for elder care modes has yielded varied results across different countries and cities, influenced by diverse factors. The significance of such factors hinges on the distinct development levels of elder care services, resource availability, and understanding of elder care systems. Methodological differences, including statistical approaches, sample sizes, definitions of dependent variables, and selection of independent variables, further contribute to the variability in findings. Existing studies on elder care preferences present several notable limitations. First, they predominantly focus on economically developed regions in Eastern and Central China, largely neglecting underdeveloped areas in Western China. Due to significant demographic and systemic differences in elder care, the findings from these studies may not be directly applicable to less developed regions. Second, the sample sizes of elderly participants in these studies are often limited, typically around 1000, which may not adequately represent the broader population’s preferences. Lastly, most research employs binary logistic regression models to analyse preferences for a single elder care mode, with few studies examining multiple care modes as dependent variables. This narrow analytical approach may fail to capture the complexity of elderly individuals' preferences, reducing the objectivity and comprehensiveness of the research findings.

As an essential gateway to the Silk Road Economic Belt on the Belt and Road Initiative, Gansu Province is located in the West of China. Despite its strategic location, Gansu is characterised by a relatively underdeveloped economy compared to other regions of China. According to the National Bureau of Statistics, the gross domestic product (GDP) per capita of China was Chinese renminbi (RMB) 89 358 in 2023 [20]. Notably, Gansu Province reported a per capita GDP of RMB 47 867, ranking last among all provinces in the country [21]. Gansu province began transitioning into an aging society in 2005 and is currently experiencing a period of rapid demographic aging. By the end of 2023, the population aged 65 and over had reached 3 364 800, accounting for 13.65% of the total population (**Figure 1**) [22]. Between 2000 and 2023, the number of elderly individuals aged 65 and above nearly tripled, highlighting the escalating severity of population aging in the province. In addition to this demographic shift, Gansu faces unique challenges exacerbated by factors such as low temperatures, high altitudes, and underdeveloped economic conditions. These contribute to a high prevalence of chronic diseases among the elderly, including hypertension, arthritis, and rheumatism. The province also experiences significant rural-to-urban migration, resulting in a loss of young labour and leaving many elderly individuals in rural areas without adequate caregiving support. This combination of demographic aging, high rates of chronic illness, and a shortage of young labour contributes to the pressing elder care challenges in Gansu Province, placing considerable strain on the region's elder care system.

**Figure 1:**
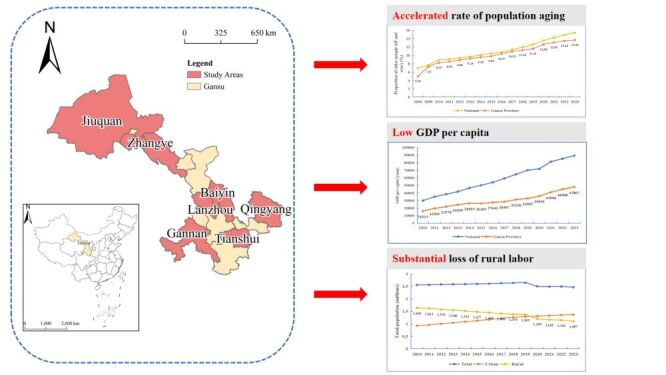
Aging pressure in Gansu province. GDP – gross domestic product.

In July 2023, we conducted a comprehensive questionnaire survey to assess the willingness of the elderly population regarding elder care modes in Gansu Province. The survey encompassed a wide geographical scope, covering seven cities, 24 counties, and 255 villages within the province, aiming to provide a comprehensive understanding of the current elder care situation in the province. The findings offer valuable insights that can guide the development and implementation of elder care service systems in underdeveloped regions of Western China.

## METHODS

### Sampling and participants

**Figure 2:**
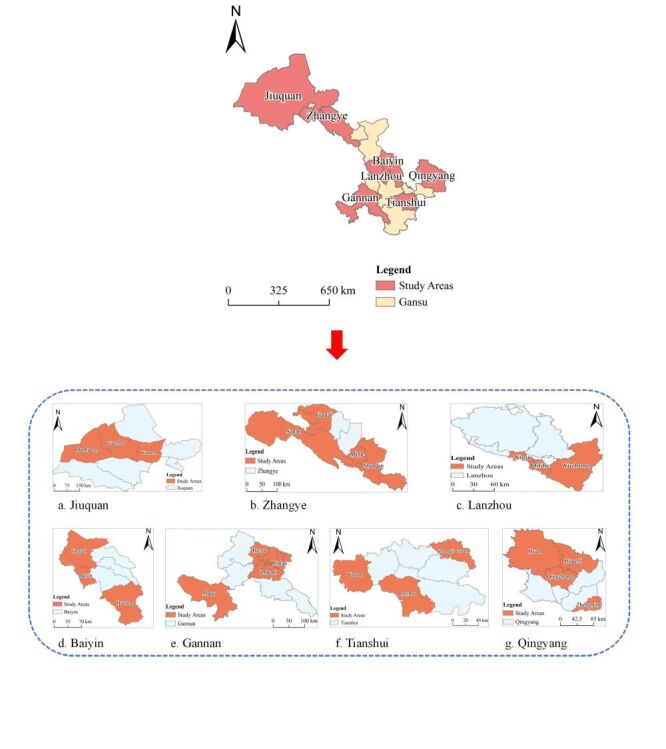
The geographical location of the study area. **Panel A.** Jiuquan. **Panel B.** Zhangye. **Panel C.** Lanzhou. **Panel D.** Baiyin. **Panel E.** Gannan. **Panel F.** Tianshui. **Panel G.** Qingyang.

In China, administrative divisions consist of provinces, cities, counties, townships and villages. This hierarchical structure forms the foundational framework for the Chinese Government's administrative system and served as the basis for the sampling process employed in this study. We utilised the multistage stratified cluster random sampling method to execute a cross-sectional survey in 24 counties encompassing seven cities in Gansu Province. The sampling procedure comprised a four-step scheme. First, we selected seven out of the 14 cities in Gansu Province based on factors such as ethnicity, geographic location, economic status, and total population. Second, we chose 24 counties based on their GDP, with rankings compiled using the per capita GDP of Gansu Province in 2021. From these rankings, we selected counties representing the highest, lowest, and intermediate GDP levels (Table S1 in the **Online Supplementary Document**). Third, we applied a 25% sampling ratio to the 337 townships in the selected counties, resulting in the random selection of 85 townships. Finally, we randomly chose three villages from each township, bringing the total number of villages to 255 (**Figure 2**, Panels A–G).

We distributed the questionnaires in proportion to the population size of the chosen districts (counties). Criteria for participant inclusion were as follows:

1) aged 60 years or older;

2) residing in the region for six months or more;

3) capable of engaging in logical discourse in the absence of any discernible cognitive impairment, severe illness, or terminal illness, as well as visual or auditory impairments caused by a variety of factors.

Before the survey, we gave all participants a brief explanation of the study's objectives and asked them to sign a consent form. We conducted the survey consistently from 5 July to 10 August 2023. We distributed a total of 20 000 questionnaires to participants.

### Instruments

The research utilised a self-administered questionnaire consisting of the following five sections (Appendix S1 in the **Online Supplementary Document**):

Section 1 comprised the socioeconomic and demographic attributes of the participants, such as registered residence, age, region, gender, ethnicity, marital status, and level of education.

Section 2 items measured the present health status of the participants, including whether suffering from chronic diseases and self-assessed health status of respondents. We utilised the number of participants afflicted with the chronic disease to assess its status.

Section 3 centred on family and social security status of the participants, including medical and endowment insurance enrolment, yearly income (RMB), number of children, relationship with children, and living arrangement. It should be noted that in China, the income sources for the elderly are primarily comprised of three components: personal labour income, family transfer income, and social security income. An analysis of data from China's Seventh National Population Census in 2020 reveals that pensions have emerged as the primary source of income for the elderly in China [23]. As of 2024, the minimum monthly pension standard for the elderly in Gansu Province is set at RMB 148, translating to an annual minimum of RMB 1776 [24]. Consequently, for the purposes of this study, we established the minimum threshold for annual income at RMB 2000 to ensure analytical rigor and relevance.

Section 4 evaluated the respondents' accessibility to health and medical resources, including factors such as the walking time from current residence to the nearest health care centre. We also incorporated an assessment of contentment with health services at the residence.

Section 5 measured the responses regarding attitudes towards living in a nursing home, and who should be relied on for elderly care. In addition, we also included an assessment of respondents' knowledge of the combination of medical and elderly care.

The dependent variable in this research was the elderly' willingness to select the elderly care mode; we assessed it through a solitary inquiry: 'Out of all the elderly care modes available, which one would you prefer to select?' (with potential answers as follows: 1 = home care, 2 = community care, 3 = institutional care, 4 = mutual care, 5 = smart care) (Table S2 in the **Online Supplementary Document**).

### Quality control

Graduate students from Lanzhou University and Gansu University of Chinese Medicine, who had received training, conducted the surveys. One hundred students from the Gansu Health Vocational College were also present. We compiled a manual containing suggestions on how to approach each query. Before the formal distribution of the questionnaire, we conducted a pre-survey with a group of one hundred retired residents who had been chosen by means of convenience sampling. Subsequently, we made appropriate revisions to the questionnaire based on the pre-survey results (Appendix S2 in the **Online Supplementary Document**). We carefully designed the procedure to ensure comprehensive data collection within feasible time constraints, with each survey taking an average of 20 minutes to complete. We designated all questions as required items, and excluded any questionnaires with missing responses from the analysis, ensuring completeness. Additionally, we conducted a thorough item-by-item review for every response to verify clarity. This process ensured that respondents had selected clear and definitive options, without any ambiguous markings or ticks. To further validate the data, we applied statistical methods, including frequency analysis and outlier detection. For continuous variables, such as age, we checked for extreme or logically inconsistent values, such as ages exceeding the known limits of the human lifespan. For categorical variables, we assessed the distribution of responses to ensure consistency and reasonableness. We implemented these measures to maintain the integrity and validity of the collected data.

### Data analysis

We conducted the statistical analysis using SPSS 27.0 (IBM Corp., Armonk, NY, USA). We used frequencies and percentages to describe the sociodemographic parameters as well as the responses to each question. We employed the Kruskal-Wallis test to evaluate differences among ordered categorical variables, and utilised the χ^2^ test to assess disparities between unordered categorical variables. We implemented multinomial logistic regression to examine the influencing factors, as it is the most suitable statistical model for categorical dependent variables. A confirmation of statistical significance regarding the differences would occur if *P* < 0.05.

## RESULTS

**Figure 3:**
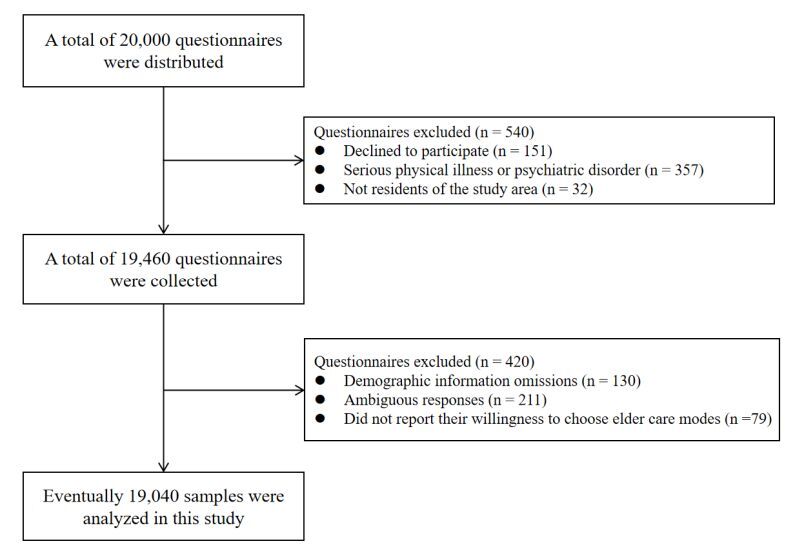
The flowchart diagram for questionnaire collection.

We distributed a total of 20 000 questionnaires for this study, and successfully collected 19 460 of them (151 individuals refused to participate, 357 were unable to complete the questionnaire due to serious physical illnesses or psychiatric disorders, and 32 were excluded because they were not residents of the study area). Following a meticulous review by the investigators, we deemed 19 040 questionnaires valid for inclusion in the subsequent analysis. Specifically, we excluded 130 questionnaires due to missing demographic information, rejected 211 because of ambiguous answers, and disqualified an additional 79 for not reporting their willingness to choose elder care modes. Consequently, the percentage of valid questionnaires was 97.8% (**Figure 3**).

### Characteristics of the participants

The majority of the 19 040 valid samples of elderly individuals consisted of females (50.1%), aged 70–79 years (50.4%), residing in rural areas (57.6%), and Han nationality (95.7%). Sixty-three point nine percent had an elementary school education or less. Forty-five point nine percent of the individuals had two children, 14 951 (78.5%) were married, and 15 314 (80.4%) did not live alone. Seven thousand five hundred and seventeen individuals (39.5%) had an annual income below RMB 2000. Seventy-seven percent were affected by chronic disease. Seven thousand seven hundred eighty-four individuals (40.9%) self-assessed their health status as 'good', while 2785 individuals (14.6%) self-assessed their health status as 'poor'. Furthermore, 18 290 (96.1%) of the participants were enrolled in medical insurance, and 18 195 (95.6%) were enrolled in endowment insurance.

### The elderly's willingness to choose the elderly care mode

Among the 19 040 valid responses, 16 860 (88.55%) preferred home care, 953 (5.01%) opted for community care, 953 (5.01%) chose institutional care, 206 (1.08%) preferred mutual care, and 68 (0.36%) selected smart care.

### Participants’ expectations of elderly services

The proportion of elderly individuals expecting to receive medical care is 49.0%. Additionally, 27.4% anticipated receiving health education, and 26.8% anticipated receiving leisure and amusement. Following that came psychiatric counselling (20.1%), daytime care (15.7%), domestic service (13.4%), re-learning service (3.6%), legal assistance (2.7%), and other services (0.6%).

### Univariate analysis of the elderly's willingness to choose the elderly care mode

The respondents' willingness towards selecting the mode of elderly care differed across several demographic factors, including age (*P* < 0.001), region (*P* < 0.001), ethnicity (*P* = 0.024), educational level (*P* < 0.001), whether suffering from chronic diseases (*P* < 0.001), self-assessed health status (*P* < 0.001), medical insurance enrolment (*P* < 0.001), endowment insurance enrolment (*P* < 0.001), yearly income (RMB) (*P* < 0.001), number of children (*P* < 0.001), relationship with children (*P* < 0.001), living arrangement (*P* < 0.001), walking time from current residence to the nearest health care centre (*P* = 0.002), satisfaction with the health service at the place of residence (*P* < 0.001), who should be relied on for elderly care? (*P* < 0.001), attitude towards living in a nursing home (*P* < 0.001), and knowledge for the combination of medical and elderly care (*P* < 0.001). On the contrary, no statistically significant relationship between different genders (*P* = 0.609), registered residence (*P* = 0.306), and marital status (*P* = 0.472) displays with the elderly's willingness to choose the elderly care mode (**Table 1**).

**Table 1:** Differences in characteristics among participants choosing home care, community care, institutional care, mutual care and smart care

Factors	Total (n = 19 040)		Home care (n = 16 860)		Community care (n = 953)		Institutional care (n = 953)		Mutual care (n = 206)		Smart care (n = 68)		χ^2^/H	*P*-value
	**n**	**%**	**n**	**%**	**n**	**%**	**n**	**%**	**n**	**%**	**n**	**%**		
**Demographic characteristics**														
Gender													2.701	0.609
*Male*	9498	49.88	8399	49.82	484	50.79	484	50.79	94	45.63	37	54.41		
*Female*	9542	50.12	8461	50.18	469	49.21	469	49.21	112	54.37	31	45.59		
Registered residence													4.819	0.306
*Rural*	10 960	57.56	9728	57.70	537	56.35	528	55.40	122	59.22	45	66.18		
*Urban*	8080	42.44	7132	42.30	416	43.65	425	44.60	84	40.78	23	33.82		
Region													77.088	<0.001
*Eastern (Qingyang and Tianshui)*	6409	33.66	5828	34.57	244	25.60	261	27.39	58	28.16	18	26.47		
*Central (Lanzhou, Baiyin and Gannan)*	9118	47.89	7927	47.02	552	57.92	512	53.73	93	45.15	34	50.00		
*Western (Jiuquan and Zhangye)*	3513	18.45	3105	18.42	157	16.47	180	18.89	55	26.70	16	23.53		
Age													39.845	<0.001
*≥90*	170	0.89	151	0.90	9	0.94	8	0.84	1	0.49	1	1.47		
*80–89*	2552	13.40	2251	13.35	137	14.38	138	14.48	18	8.74	8	11.76		
*70–79*	9588	50.36	8605	51.04	451	47.32	398	41.76	98	47.57	36	52.94		
*60–69*	6730	35.35	5853	34.72	356	37.36	409	42.92	89	43.20	23	33.82		
Ethnicity													11.206	0.024
*Han*	18 228	95.74	16 113	95.57	928	97.38	924	96.96	197	95.63	66	97.06		
*Minority*	812	4.26	747	4.43	25	2.62	29	3.04	9	4.37	2	2.94		
Educational level													431.405	<0.001
*Bachelor degree or above*	233	1.22	198	1.17	16	1.68	15	1.57	3	1.46	1	1.47		
*Junior college*	543	2.85	464	2.75	25	2.62	43	4.51	7	3.40	4	5.88		
*Senior high school or technical secondary school*	2438	12.80	2054	12.18	170	17.84	165	17.31	35	16.99	14	20.59		
*Junior high school*	3662	19.23	3108	18.43	253	26.55	243	25.50	45	21.84	13	19.12		
*Elementary school and below*	12 164	63.89	11 036	65.46	489	51.31	487	51.10	116	56.31	36	52.94		
Marital status													11.673	0.472
*Unmarried*	174	0.91	151	0.90	6	0.63	13	1.36	4	1.94	0	0.00		
*Married*	14 951	78.52	13 245	78.56	748	78.49	742	77.86	163	79.13	53	77.94		
*Divorced*	217	1.14	185	1.10	15	1.57	14	1.47	1	0.49	2	2.94		
*Widowed*	3698	19.42	3279	19.45	184	19.31	184	19.31	38	18.45	13	19.12		
**Health status**														
Whether suffering from chronic diseases													40.566	<0.001
*No*	4382	23.01	3962	23.50	217	22.77	155	16.26	27	13.11	21	30.88		
*Yes*	14 658	76.99	12 898	76.50	736	77.23	798	83.74	179	86.89	47	69.12		
Self-assessed health status													72.630	<0.001
*Very poor*	383	2.01	325	1.93	20	2.10	30	3.15	8	3.88	0	0.00		
*Poor*	2785	14.63	2448	14.52	145	15.22	156	16.37	29	14.08	7	10.29		
*Fair*	6407	33.65	5507	32.66	426	44.70	360	37.78	83	40.29	31	45.59		
*Good*	7784	40.88	7023	41.65	317	33.26	347	36.41	70	33.98	27	39.71		
*Very good*	1681	8.83	1557	9.23	45	4.72	60	6.30	16	7.77	3	4.41		
**Family and social security**														
Medical insurance enrolment													23.205	<0.001
*No*	750	3.94	702	4.16	18	1.89	19	1.99	7	3.40	4	5.88		
*Yes*	18 290	96.06	16 158	95.84	935	98.11	934	98.01	199	96.60	64	94.12		
Endowment insurance enrolment													62.074	<0.001
*No*	845	4.44	753	4.47	21	2.20	37	3.88	30	14.56	4	5.88		
*Yes*	18 195	95.56	16 107	95.53	932	97.80	916	96.12	176	85.44	64	94.12		
Yearly income (RMB)													237.443	<0.001
*≤2000*	7517	39.48	6888	40.85	248	26.02	312	32.74	56	27.18	13	19.12		
*2001–10 000*	4531	23.80	4051	24.03	206	21.62	208	21.83	55	26.70	11	16.18		
*10 001–30 000*	2975	15.63	2588	15.35	189	19.83	146	15.32	38	18.45	14	20.59		
*30 001–50 000*	2559	13.44	2098	12.44	215	22.56	189	19.83	38	18.45	19	27.94		
*>50 000*	1458	7.66	1235	7.33	95	9.97	98	10.28	19	9.22	11	16.18		
Number of children													479.738	<0.001
*0*	255	1.34	155	0.92	21	2.20	76	7.97	2	0.97	1	1.47		
*1*	2712	14.24	2144	12.72	261	27.39	238	24.97	45	21.84	24	35.29		
*2*	8734	45.87	7711	45.74	453	47.53	433	45.44	105	50.97	32	47.06		
*≥3*	7339	38.55	6850	40.63	218	22.88	206	21.62	54	26.21	11	16.18		
Relationship with children													145.291	<0.001
*Very poor*	54	0.28	46	0.27	3	0.31	4	0.42	1	0.49	0	0.00		
*Poor*	253	1.33	184	1.09	38	3.99	28	2.94	3	1.46	0	0.00		
*Fair*	2972	15.61	2480	14.71	210	22.04	229	24.03	35	16.99	18	26.47		
*Good*	11 397	59.86	10 229	60.67	541	56.77	460	48.27	134	65.05	33	48.53		
*Very good*	4109	21.58	3766	22.34	140	14.69	156	16.37	31	15.05	16	23.53		
Living arrangement													19.903	<0.001
*Not alone*	15 314	80.43	13 609	80.72	759	79.64	717	75.24	170	82.52	59	86.76		
*Alone*	3726	19.57	3251	19.28	194	20.36	236	24.76	36	17.48	9	13.24		
**Accessibility of medical and health resources**														
Walking time from current residence to the nearest health care centre													23.854	0.002
*Not have*	315	1.65	289	1.71	10	1.05	13	1.36	3	1.46	0	0.00		
*≤15 min*	10 100	53.05	8984	53.29	487	51.10	484	50.79	95	46.12	50	73.53		
*>15 min*	8625	45.30	7587	45.00	456	49.85	456	48.85	108	52.43	18	26.47		
**Satisfaction with the health service at the place of residence**													116.287	<0.001
Very dissatisfied	88	0.46	72	0.43	5	0.52	8	0.84	3	1.46	0	0.00		
Dissatisfied	285	1.50	220	1.30	27	2.83	33	3.46	4	1.94	1	1.47		
Fair	2709	14.23	2193	13.01	281	29.49	178	18.68	40	19.42	17	25.00		
Satisfied	13 017	68.37	11 777	69.85	491	51.52	564	59.18	141	68.45	44	64.71		
Very satisfied	2941	15.45	2598	15.41	149	15.63	170	17.84	18	8.74	6	8.82		
**The concept of elderly care**														
Who should be relied on for elderly care?													487.019	<0.001
*Oneself*	6900	36.24	6074	36.03	341	35.78	361	37.88	86	41.75	38	55.88		
*Children*	10 464	54.96	9539	56.58	461	48.37	351	36.83	93	45.15	20	29.41		
*Government*	1676	8.80	1247	7.40	151	15.84	241	25.29	27	13.11	10	14.71		
**Attitude towards living in a nursing home**													1749.937	<0.001
Opposition	10 842	56.94	10 388	61.61	255	26.76	126	13.22	57	27.67	16	23.53		
Unclear	3060	16.07	2639	15.65	218	22.88	134	14.06	64	31.07	5	7.35		
Support	5138	26.99	3833	22.73	480	50.37	693	72.72	85	41.26	47	69.12		
**Knowledge for the combination of medical and elderly care**													764.772	<0.001
Never heard	12 482	65.56	11 566	68.60	358	37.57	435	45.65	104	50.49	19	27.94		
Heard but not understood	3914	20.56	3326	19.73	251	26.34	261	27.39	56	27.18	20	29.41		
Have gained some understanding	2341	12.30	1777	10.54	289	30.33	208	21.83	43	20.87	24	35.29		
Know well	303	1.59	191	1.13	55	5.77	49	5.14	3	1.46	5	7.35		

### Multivariate logistic regression analysis of the influencing factors on the elderly's willingness to choose the elderly care mode

We employed multivariate logistic regression to analyse the factors influencing the elderly care mode choices among elderly in Gansu Province. We set institutional care as the reference category (Tables S2–3 in the **Online Supplementary Document**).

**Figure 4:**
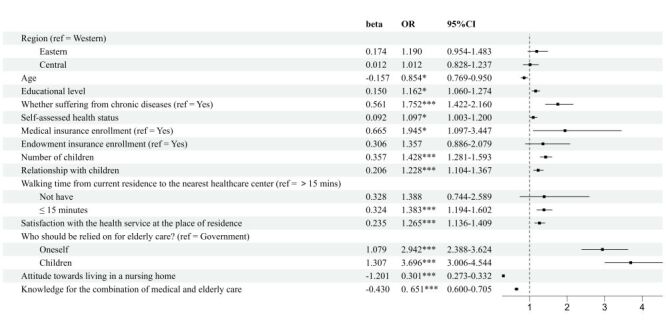
Logistic regression results of the influencing factors on the elderly's willingness to choose the elderly care mode (home care vs institutional care). CI – confidence interval, OR – odds ratio.

Elderly individuals who have a lower level of education (odds ratio (OR) = 1.162; 95% CI = 1.060–1.274, *P* = 0.001), do not suffer from chronic illnesses (OR = 1.752; 95% CI = 1.422–2.160, *P* < 0.001), report good self-assessed health (OR = 1.097; 95% CI = 1.003–1.200, *P* = 0.043), lack of medical insurance (OR = 1.945; 95% CI = 1.097–3.447, *P* = 0.023), a greater number of children (OR = 1.428; 95% CI = 1.281–1.593; *P* < 0.001), positive relationships with their children (OR = 1.228; 95% CI = 1.104–1.367, *P* < 0.001), residence within a 15-minute walking distance to the nearest health care facility (OR = 1.383; 95% CI = 1.194–1.602, *P* = <0.001), high satisfaction with the services provided by the medical centre (OR = 1.265; 95% CI = 1.136–1.409, *P* < 0.001), and believe that elder care should rely on themselves (OR = 2.942; 95% CI = 2.388–3.624, *P* < 0.001) or their children (OR = 3.696; 95% CI = 3.006–4.544; *P* < 0.001), exhibit a higher willingness for choosing the home care mode. Conversely, those who are relatively younger in age (OR = 0.854; 95% CI = 0.769–0.950, *P* = 0.004), hold positive attitudes towards residing in nursing homes (OR = 0.301; 95% CI = 0.273–0.332, *P* < 0.001), and possess a greater knowledge for the combination of medical and elderly care (OR = 0.651; 95% CI = 0.600–0.705, *P* < 0.001), demonstrate a lower willingness for choosing the home care mode (**Figure 4**).

**Figure 5:**
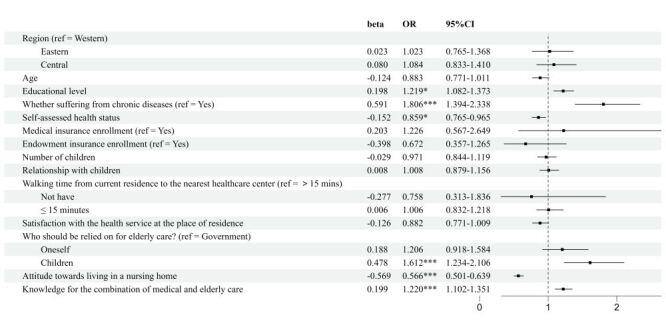
Logistic regression results of the influencing factors on the elderly's willingness to choose the elderly care mode (community care vs institutional care). CI – confidence interval, OR – odds ratio.

Elderly individuals with lower level of education (OR = 1.219; 95% CI = 1.082–1.373, *P* = 0.001), who do not suffer from chronic illnesses (OR = 1.806; 95% CI = 1.394–2.338, *P* < 0.001), believe that elderly care should be reliant on their children (OR = 1.612; 95% CI = 1.234–2.106, *P* < 0.001), and possess a greater knowledge for the combination of medical and elderly care (OR = 1.220; 95% CI = 1.102–1.351, *P* < 0.001), exhibit a stronger willingness for choosing the community care. On the contrary, those who report good self-assessed health (OR = 0.859; 95% CI = 0.765–0.965, *P* = 0.010), and hold a more positive attitude towards residing in nursing homes (OR = 0.566; 95% CI = 0.501–0.639, *P* < 0.001), demonstrate a lower willingness for choosing the community care (**Figure 5**).

**Figure 6:**
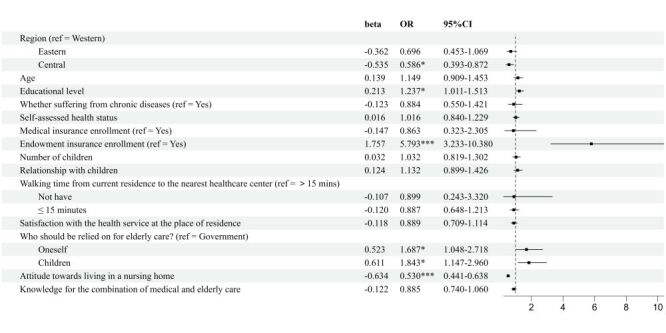
Logistic regression results of the influencing factors on the elderly's willingness to choose the elderly care mode (mutual care vs institutional care). CI – confidence interval, OR – odds ratio.

Elderly individuals with lower educational level (OR = 1.237; 95% CI = 1.011–1.513, *P* = 0.038) and lack endowment insurance (OR = 5.793; 95% CI = 3.233–10.380, *P* < 0.001), who believe that elderly care should rely on themselves (OR = 1.687; 95% CI = 1.048–2.718, *P* = 0.031) or their children (OR = 1.843; 95% CI = 1.147–2.960, *P* = 0.001), exhibit a higher willingness for choosing the mutual care. Conversely, elderly people in the central region of Gansu Province (OR = 0.586; 95% CI = 0.393–0.872, *P* = 0.008), as well as those who hold a more positive attitude towards residing in nursing homes (OR = 0.530; 95% CI = 0.441–0.638, *P* < 0.001), demonstrate a lower willingness for choosing the mutual care (**Figure 6**).

**Figure 7:**
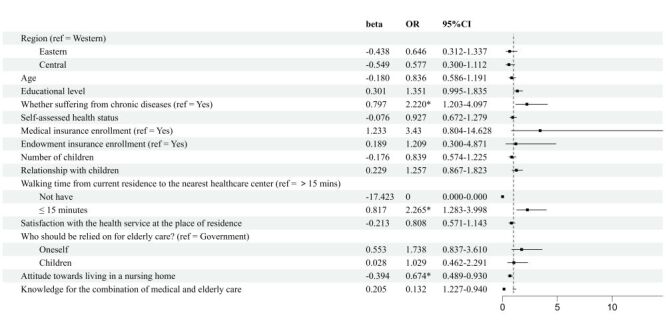
Logistic regression results of the influencing factors on the elderly's willingness to choose the elderly care mode (smart care vs institutional care). CI – confidence interval, OR – odds ratio.

Elderly individuals who are not afflicted with chronic illnesses (OR = 2.220; 95% CI = 1.203–4.097, *P* = 0.011), reside within a 15-minute distance from the nearest medical centre (OR = 2.265; 95% CI = 1.283–3.998, *P* = 0.005) exhibit a higher willingness for choosing the smart care mode. However, those with more positive attitudes towards residing in nursing homes (OR = 0.674; 95% CI = 0.489–0.930, *P* = 0.016) demonstrate a lower willingness for choosing the smart care mode (**Figure 7**).

## DISCUSSION

We adopted the multistage stratified cluster random sampling method to investigate the willingness of 19 040 elderly individuals in seven cities of Gansu Province, an underdeveloped area in Western China. The research results show that the elderly in Gansu Province preferred family care (88.55%), community care (5.01%), and institutional care (5.01%), which is consistent with previous research results. Family elder care remains the preferred option for most elderly individuals, both in China and globally. This preference is rooted in the fact that family is the most fundamental and familiar social unit, and people generally favour receiving support at home rather than in institutional settings. In China, traditional cultural values, such as the concepts of ‘raising children to ensure support in old age’ and ‘Filial piety’, are deeply ingrained, reinforcing the expectation that elderly individuals receive care and emotional support from their families. Additionally, many elderly individuals are accustomed to their long-established community environments, neighbourhood relationships, and social networks, which further contribute to their willingness for remaining at home to receive elder care [25]. However, the findings of the present study indicate that the proportion of elderly individuals in Gansu Province who preferred home care differs from that in other regions of China, such as Beijing (78%) [18], Chongqing (85.4%) [16], and Xiamen (86.37%) [25]. Further, one possible reason for the willingness for home elder care in Gansu Province is its relatively low level of economic development compared to other regions. Both urban and rural residents in Gansu have a lower per capita discretionary income, which limits the elderly's ability to afford socialised elder care services. In 2023, the disposable income per capita of residents in Gansu Province ranked last in China [22]. Another reason is the rapid growth of the elderly population in Gansu Province, which is experiencing a deepening aging trend and an increasing prevalence of age-related disabilities. Despite this, by the end of 2020, the proportion of nursing beds in elder care institutions in Gansu was only 42%, significantly limiting the availability of institutional care services. As a result, the growing elder care needs of the population remain unmet, further reinforcing the reliance on home care, especially in the face of limited institutional options [26].

With the development of the social economy and the change in family structure, several new modes of elder care, such as mutual care and smart care, have gradually emerged. The results of the present study show that the proportions of the elderly in Gansu province who preferred mutual care and smart care accounted for 1.08 and 0.36%, respectively. This indicates that, although the proportion is still low, the demand for alternative elder care modes in Gansu Province is gradually emerging. In the future, financial subsidies and preferential policies from government agencies will likely play a key role in supporting family-based care models. These measures will be essential to ensuring that the needs of the majority of elderly individuals who prefer home care are adequately met, while also addressing the growing demand for more diverse elder care options in the province. At the same time, relevant government departments should prioritise publicity and education efforts to raise awareness and understanding of new elder care modes. By strengthening outreach and improving public knowledge, they can enhance the elderly's sense of identity and acceptance of these alternatives. This would help shift the elderly's preference for care from a singular reliance on home-based care to more diversified care models that involve shared responsibilities across government, society, and the elderly themselves. Such initiatives would facilitate a more balanced and sustainable approach to elder care in the region.

Based on pertinent literature, we categorised elderly services into the following domains: medical care, health education, daytime care, domestic assistance, recreational and entertainment, psychological counselling, legal aid, and re-learning services [5,27,28]. The present findings reveal that medical care is the service with the greatest demand among the elderly population, aligning with the conclusions of previous research. As a distinctive elder care model in China, the integration of medical and elderly care has been empirically demonstrated in numerous studies to effectively address the simultaneous medical and care needs of the aging population. This approach enhances the efficiency of utilising both health care and elderly care resources. Given the demographic challenges posed by an aging society, it is crucial for China to actively promote the integration of medical and elderly care as a key strategy to meet the growing needs of the elderly population and improve overall care delivery in the future.

### Personal characteristic factors

The present findings indicate that age, region, and education level affect the elderly's willingness for elder care. These three factors are among the most frequently cited determinants in existing literature on the demographic characteristics that affect elder care choices. Regarding age, younger elderly individuals tend to be more inclined toward institutional care, which aligns with the findings of Tan (2020) [29] and Wu (2020) [30]. This preference can be attributed to generational differences, as younger elderly individuals grew up during pivotal historical periods, such as the restoration of the college entrance examination system and China's reform and opening-up. These experiences have shaped their cultural education, economic prospects, awareness of rights, and sense of agency, leading to a greater enthusiasm for socialised elder care services [31]. However, several studies have reported that older elderly individuals are more likely to require both physical and psychological care. Considering that families cannot meet most of these needs, the willingness to accept institutional elder care increases with age [3,32]. Therefore, in the future, it is necessary to strengthen the propaganda of institutional elder care and improve the elderly's awareness of professional nursing services in elder care institutions.

Geographically, elderly individuals from the central region of Gansu Province, specifically Lanzhou and Baiyin, show less willingness to choose mutual support elder care. Research indicates that in more economically underdeveloped areas, where there is a significant outflow of young and middle-aged labour and limited family-based elder care resources, the demand for mutual support elder care tends to be higher [33]. The central regions investigated in the present study were Lanzhou and Baiyin. These two cities, with relatively higher levels of social and economic development compared to other areas in Gansu Province, show that the elderly are less inclined to choose mutual support elder care. This may be due to the availability of alternative care options, such as home or institutional care, which are more accessible in these regions. In terms of education level, the findings reveal that elderly individuals with lower education levels are more likely to prefer home care, community care, or mutual support care, as opposed to institutional care. This observation aligns with findings from previous studies [34,35]. Generally, elderly individuals with higher education levels tend to have more stable incomes, better living conditions, and more modern attitudes toward elder care. They are typically more open to new care models and less dependent on their children for daily support. As a result, they are more likely to seek formal care in elder care institutions, driven by a greater emphasis on health and quality of life [36].

### Health status factors

In terms of health status, this study found that the presence of chronic diseases and self-rated health status significantly influence the elderly's preferences for long-term care options. Self-assessed health refers to an individual's subjective evaluation and expectations regarding their own health status. Existing research has demonstrated that, for the elderly population, self-assessed health is an effective measure for assessing subjective health status [37,38]. Self-assessed health represents an individual's overall judgment of their health status, integrating subjective and objective health information based on their perceptions of physical, psychological, and social adaptation aspects [39]. Consequently, elderly individuals with better self-rated health are likely to be in a relatively healthy state both physically and psychologically. This state is a comprehensive reflection of various factors, including the maintenance of physical function, a positive psychological state, and active social engagement.

Specifically, older adults who were free from chronic conditions and reported better self-rated health were more likely to choose home or community-based elder care. The elderly with chronic diseases and poor self-assessed health status were more likely to accept formal institutional elder care, which is consistent with many existing research results [6,12]. The reason may be that with the increase in the age of the elderly, their physical function gradually weakens, and the sickness probability and disability degree of chronic diseases increases. At the same time, compared with other regions, the characteristics of high average altitude and low temperature in Gansu Province have led to a higher proportion of the elderly suffering from chronic diseases such as hypertension, arthritis, or rheumatism. Elderly individuals with poor health require daily assistance and professional support in areas such as disease diagnosis and treatment, chronic disease management, health consultation, and rehabilitation nursing [36].

### Family and social security factors

Regarding family and social security factors, the number of children, the quality of the relationship with children, and the decision to purchase medical and endowment insurance were found to influence the elderly's willingness for elder care options. Elderly with more children and better relationships with them were more likely to prefer home elder care, which is consistent with previous research [6,11]. Traditionally, spouses and children have been the primary sources of social support for the elderly, and a larger number of children often translates to more caregiving resources and greater security for the elderly. Additionally, when children provide more emotional or practical care, the likelihood of choosing home care increases [40]. However, due to China’s family planning policies and rapid socio-economic changes, family structures have shifted, with smaller families and higher rates of childlessness becoming more common. The '421 family model' (four grandparents, two parents, one child) has emerged as the norm, weakening the traditional family care model and increasing caregiving pressure on younger generations [8]. As a result, children are less able to provide comprehensive care, especially when elderly individuals suffer from disabilities or chronic conditions [11].

Further, elderly individuals with medical insurance are more inclined to choose institutional care, as insurance helps mitigate the higher costs associated with care institutions, particularly for those with poor health [19]. Elderly individuals without endowment insurance are more likely to prefer mutual care modes. This may be due to the role of endowment insurance in ensuring the elderly's basic livelihood and reducing economic strain. In rural areas, where endowment insurance is a crucial financial safeguard, it provides a stable income that meets the elderly's basic needs [41]. Studies suggest that the economic security provided by endowment insurance can act as a 'substitution effect', giving the elderly a greater sense of security and satisfaction, which reduces their need for other forms of elder care [41]. As a result, those with endowment insurance are less likely to seek alternative care options, feeling more financially secure in their old age.

Furthermore, although the annual income of the elderly was not found to be a significant factor influencing their willingness to choose an elder care mode in this study, it is noteworthy that 39.5% of the elderly participants reported an annual income below RMB 2000. Based on the data from the Fifth China Urban and Rural Elderly Living Conditions Sample Survey, the annual per capita income of elderly individuals in China in 2021 was RMB 32 027.4. Specifically, the urban elderly population had an annual per capita income of RMB 47 270.8, and the rural elderly population had an annual per capita income of RMB 14 105.4 [42]. These figures indicate that the annual income of the elderly in Gansu Province is significantly lower than the Chinese average.

### Availability of medical and health resources

Regarding the availability of medical and health resources, the distance from the elderly's residence to the local health service centre and their satisfaction with the services provided by the centre were found to influence their willingness for elder care. Specifically, elderly individuals who lived within a 15-minute walk from the local health service centre and were more satisfied with the services offered were more likely to choose home elder care, a finding consistent with previous research [18,43]. In China, community health service centres are primarily responsible for delivering public health and essential medical care services to residents, including health management services tailored to the elderly to address their basic health needs [15]. Generally, when elderly individuals live near a local health service centre and report high satisfaction with the care they receive, it indicates that they can access timely professional health care services within their community, which supports their preference for receiving care at home. In addition, influenced by traditional Chinese cultural values, many elderly individuals prefer to live in a familiar family environment for elder care, making them more inclined to choose home care [15]. This preference underscores the importance of integrating existing medical and elder care resources within the community. Strengthening medical services through models like family doctor systems and cloud-based medical platforms can help meet the professional and personalised health care needs of the elderly at home. However, evidence from cities like Nanchang and Chongqing highlights a shortage of medical resources in China’s current family elder care system. A key issue is the lack of effective integration between medical and elder care services, which hampers the ability to meet the health care needs of elderly individuals [44,45]. In economically underdeveloped regions of Western China, the problem is even more pronounced due to the relatively low level of economic development, an unreasonable distribution of medical resources, and regional disparities in health care access. This has led to an imbalance between the supply and demand for medical services for the elderly, especially when compared to the more developed Eastern regions.

### The concepts of elder care

Regarding the elderly's perceptions of elder care, particularly in terms of who should provide care, their attitudes toward nursing homes, and their knowledge of the combination of medical and elderly care, significantly influenced their willingness to choose certain elder care modes. Elderly individuals who believed that they should rely on themselves or their children for care tended to be less inclined to choose institutional care, while those with a more positive attitude toward staying in nursing homes were more likely to opt for institutional care. Typically, elderly people who prefer to rely on themselves or their children for care are more conservative in their views, with a stronger dependence on family caregiving and a greater expectation of being cared for by their children in daily life. Previous studies, including research in Beijing, have shown that the elderly tend to hold diverse views on elder care, with a roughly 5:3:2 ratio of those who believe care should depend on the government, children, or themselves, respectively [46]. In contrast, the findings of this study show that 91.20% of the elderly in Gansu Province believe elder care should rely on themselves or their children, with only 8.80% favouring government-provided care. This indicates that the concept of elder care in Gansu is more conservative than in other regions.

This conservative mindset is a major barrier to adopting newer elder care models, such as institutional care. Many elderly individuals still prefer home care, while some children view placing their elderly parents in nursing homes as a sign of filial ingratitude. As a result, institutional elder care has not gained widespread social acceptance [6,47]. With the aging population in China becoming increasingly significant, socialised elder care modes, including institutional and community care, are becoming increasingly essential to the elder care system. To address this, government departments should promote the advantages of institutional care and other socialised care options, as well as the widespread use of such models in other countries, in an effort to shift deeply ingrained cultural perceptions.

Additionally, this study found that elderly individuals who were more aware of the combination of medical and elderly care were more likely to choose institutional or community care. The combination of medical and elderly care represents an innovative elder care mode that combines health care and daily caregiving services to address the complex needs of the elderly, including disease diagnosis and treatment, health guidance, rehabilitation, and hospice care. This model is a key component of China's current elder care policy [5]. However, many studies indicate that awareness of integrated care is still low among the elderly in China. For example, only 12% of elderly residents in Beijing, 25% in Datong, and 8.13% in Lanzhou, Gansu, are aware of this model [5,48,49]. Therefore, in the context of China's aging population and the government’s push for diversified elder care, it is essential for authorities to strengthen public awareness campaigns about the combination of medical and elderly care. This can be done through various media channels, such as television, newspapers, and community-based institutions, ensuring that information reaches both the elderly and their family members. By doing so, public recognition and acceptance of diversified elder care models can be significantly improved [19].

### Strengths and limitations

The present study has several limitations. First, we adopted a cross-sectional design from the perspective of research methodology. Thus, the survey data cannot support causal relationships between variables, and causal inferences regarding variables should be made cautiously. Moreover, with time, the health status of elderly people undergoes dynamic changes. Therefore, future research could explore the causal relationships of factors related to elderly care modes based on longitudinal or follow-up data. Second, regarding influencing factors, elderly individuals' willingness for elderly care modes are not only related to their actual needs but also to the willingness of family members (especially primary caregivers) and the availability of elderly care institution services, a relatively neglected aspect in current research. Therefore, while understanding the willingness of elderly individuals is crucial, it is equally important to consider the willingness of their family members regarding elder care, as well as the accessibility of elder care services within the community. Further, the present study relied on subjective survey assessment tools, which may introduce reporting biases, particularly in the absence of objective indicators such as annual income. To address these limitations, future research should incorporate objective data where possible and employ strategies to minimise potential biases, ensuring a more accurate and comprehensive understanding of the factors influencing elder care decisions.

Despite these limitations, the present study makes significant contributions. To the present knowledge, the study is the first to focus on elderly individuals in underdeveloped regions of Western China, delving into their willingness for elderly care modes. In the research design phase, a scientifically rigorous multistage stratified cluster random sampling method was employed to select representative and valid sample of 19 040 respondents. Second, we analysed the three main elderly care modes in China (home care, community care, and institutional care), along with two emerging modes (mutual care and smart care), as dependent variables, enabling the research findings to reflect elderly individuals' willingness for elderly care modes objectively. Finally, based on existing literature, we categorised the potential factors influencing elderly individuals' willingness for elderly care modes into five categories. These factors served as the sources of independent variables in the present study, ensuring that the selected independent variables were scientific, comprehensive, and reliable.

## CONCLUSIONS

In underdeveloped regions of Western China, home care remains the preferred option for most elderly individuals, although the demand for diversified elder care models should not be overlooked. The willingness for different types of elder care is shaped by a variety of factors, with medical care services being the most in demand. To address these needs, the government should prioritise strengthening the home care model while also developing a coordinated 'institution-community-home' framework to provide a more diversified and flexible approach to elder care. Further, we should make efforts to enhance the dissemination of relevant policies, regulations, and exemplary cases that encourage elderly individuals to reconsider traditional views on elder care, fostering greater awareness and acceptance of socialised and innovative care models. Finally, integrating existing medical and elder care resources and expanding the availability of home-based medical services will be essential to meet the specialised and personalised health care needs of elderly individuals, ensuring they can receive comprehensive care at home while addressing their diverse needs.

## Additional material


Online Supplementary Document
