# Supplementary material for: Willingness and determinants of elder care modes among elderly individuals: insights from underdeveloped regions in Western China
Source: J Glob Health. 2025 Jan 24;15:04031. doi: 10.7189/jogh.15.04031 (PMC11758468; doi:10.7189/jogh.15.04031)
Supplement: Online Supplementary Document [file jogh-15-04031-s001.pdf]

Supplementary

Table S1 Number of GDP per capita in the 24 counties sampled in 2021

Cities	County	GDP per capita (RMB: yuan)
Lanzhou	Xigu	109133
	Qilihe	75178
	Yuzhong	40887
Qingyang	Huachi	84180
	Zhengning	11280
	Qingcheng	32584
	Huanxian	35597
Tianshui	Qinzhou	36252
	Zhangjiachuan	8939
	Wushan	15194
Baiyin	Baiyin	85462
	Huining	13573
	Jingtai	29797
Zhangye	Minle	27951
	Shandan	35122
	Gaotai	38202
	Sunan	84763
Jiuquan	Yumen	161228
	Dunhuang	58623
	Guazhou	92570
Gannan	Hezuo	63408
	Lintan	17221
	Zhuoni	26555
	Maqu	39533

Data source: China County Statistical Yearbook

Table S2 Variable definition for the elderly's willingness to choose the elderly care mode

Variable Categories	Variable Name	Code
Region	X_1	Eastern = 1, Central = 2, Western = 3
Age	X_2	⩾90 = 1, 80-89 = 2, 70-79 = 3, 60-69 = 4
Ethnicity	X_3	Han = 1, Minority = 2
Educational level	X_4	Bachelor degree or above = 1, Junior college = 2, Senior high school or technical secondary school = 3, Junior high school = 4, Elementary school and below = 5
Whether suffering from chronic diseases	X_5	No = 1, Yes = 2
Self-assessed health status	X_6	Very poor = 1, Poor = 2, Fair = 3, Good = 4, Very good = 5
Medical insurance enrollment	X_7	No = 1, Yes = 2
Endowment insurance enrollment	X_8	No = 1, Yes = 2
Yearly income (RMB)	X_9	⩽ 2000 = 1, 2001- 10000 = 2, 10001- 30000 = 3, 30001 - 50000 = 4, ＞ 50000 = 5
Number of children	X_{10}	0 = 1, 1 = 2, 2 = 3, ⩾3 = 4
Relationship with children	X_{11}	Very poor = 1, Poor = 2, Fair = 3, Good = 4, Very good =5
Living arrangement	X_{12}	Not alone = 1, Alone = 2
Walking time from current residence to the nearest healthcare center	X_{13}	Not have = 1, ⩽ 15 minutes = 2, ＞ 15 minutes = 3
Satisfaction with the health service at the place of residence	X_{14}	Very dissatisfied = 1, Dissatisfied = 2, Fair = 3, Satisfied = 4, Very satisfied = 5
Who should be relied on for elderly care?	X_{15}	Oneself = 1, Children = 2, Government = 3
Attitude towards living in a nursing home	X_{16}	Opposition = 1, Unclear = 2, Support = 3
Knowledge of the combination with medical care	X_{17}	Never heard = 1, Heard but not understood = 2, Have gained some understanding = 3, Know well = 4
Which elderly care mode would you like to choose?	Y	home care = 1, community care = 2, institutional care = 3, mutual care = 4, smart care = 5

Table S3 Logistic regression analysis of the influencing factors on the elderly's willingness to choose the elderly care mode (Institutional care as a reference)

	Home care				Community care				Mutual care				Smart care			
	β	*P*	*OR*	*95%CI*	β	*P*	*OR*	*95%CI*	β	*P*	*OR*	*95%CI*	β	*P*	*OR*	*95%CI*
Region (ref = Western)																
Eastern	0.174	0.123	1.190	0.954-1.483	0.023	0.879	1.023	0.765-1.368	-0.362	0.098	0.696	0.453-1.069	-0.438	0.239	0.646	0.312-1.337
Central	0.012	0.906	1.012	0.828-1.237	0.080	0.550	1.084	0.833-1.410	-0.535	0.008	0.586*	0.393-0.872	-0.549	0.100	0.577	0.300-1.112
Age	-0.157	0.004	0.854*	0.769-0.950	-0.124	0.072	0.883	0.771-1.011	0.139	0.245	1.149	0.909-1.453	-0.180	0.320	0.836	0.586-1.191
Educational level	0.150	0.001	1.162*	1.060-1.274	0.198	0.001	1.219*	1.082-1.373	0.213	0.038	1.237*	1.011-1.513	0.301	0.054	1.351	0.995-1.835
Whether suffering from chronic diseases (ref = Yes)	0.561	< 0.001	1.752***	1.422-2.160	0.591	< 0.001	1.806***	1.394-2.338	-0.123	0.611	0.884	0.550-1.421	0.797	0.011	2.220*	1.203-4.097
Self-assessed health status	0.092	0.043	1.097*	1.003-1.200	-0.152	0.010	0.859*	0.765-0.965	0.016	0.872	1.016	0.840-1.229	-0.076	0.654	0.927	0.672-1.279
Medical insurance enrollment (ref = Yes)	0.665	0.023	1.945*	1.097-3.447	0.203	0.065	1.226	0.567-2.649	-0.147	0.769	0.863	0.323-2.305	1.233	0.096	3.430	0.804-14.628
Endowment insurance enrollment (ref = Yes)	0.306	0.160	1.357	0.886-2.079	-0.398	0.218	0.672	0.357-1.265	1.757	< 0.001	5.793***	3.233-10.380	0.189	0.790	1.209	0.300-4.871
Number of children	0.357	< 0.001	1.428***	1.281-1.593	-0.029	0.688	0.971	0.844-1.119	0.032	0.787	1.032	0.819-1.302	-0.176	0.363	0.839	0.574-1.225
Relationship with children	0.206	< 0.001	1.228***	1.104-1.367	0.008	0.911	1.008	0.879-1.156	0.124	0.292	1.132	0.899-1.426	0.229	0.227	1.257	0.867-1.823
Walking time from current residence to the nearest healthcare center (ref = ＞ 15 mins)																
Not have	0.328	0.303	1.388	0.744-2.589	-0.277	0.539	0.758	0.313-1.836	-0.107	0.873	0.899	0.243-3.320	-17.423	0.998	0.000	0.000-0.000
≤ 15 minutes	0.324	< 0.001	1.383***	1.194-1.602	0.006	0.947	1.006	0.832-1.218	-0.120	0.453	0.887	0.648-1.213	0.817	0.005	2.265*	1.283-3.998
Satisfaction with the health	0.235	< 0.001	1.265***	1.136-1.409	-0.126	0.068	0.882	0.771-1.009	-0.118	0.307	0.889	0.709-1.114	-0.213	0.229	0.808	0.571-1.143

service at the place of residence																
Who should be relied on for elderly care? (ref = Government)																
Oneself	1.079	< 0.001	2.942***	2.388-3.624	0.188	0.178	1.206	0.918-1.584	0.523	0.031	1.687*	1.048-2.718	0.553	0.138	1.738	0.837-3.610
Children	1.307	< 0.001	3.696***	3.006-4.544	0.478	< 0.001	1.612***	1.234-2.106	0.611	0.001	1.843*	1.147-2.960	0.028	0.954	1.029	0.462-2.291
Attitude towards living in a nursing home	-1.201	< 0.001	0.301***	0.273-0.332	-0.569	< 0.001	0.566***	0.501-0.639	-0.634	< 0.001	0.530***	0.441-0.638	-0.394	0.016	0.674*	0.489-0.930
Knowledge for the combination of medical and elderly care	-0.430	< 0.001	0.651***	0.600-0.705	0.199	< 0.001	1.220***	1.102-1.351	-0.122	0.185	0.885	0.740-1.060	0.205	0.132	0.132	1.227-0.940

β : Regression coefficient; *OR*: Ratio of odds; *CI:* Confidence interval; *$P < 0.05$, ***$P < 0.001$

Appendix S1 The full questionnaire used in this study

To whom it may concern:

Hello! This survey is intended to help us understand the willingness of the elderly to choose the elderly care mode and its influencing factors. The contents of all questionnaires will be kept completely confidential and only used by researchers for data analysis. All you need to do is to fill in the questionnaire based on your actual situation. Your answer will provide a valuable reference for us to draw correct conclusions. We really appreciate your kind support and contribution. Many thanks!

The following information was completed by the investigator:

Current address of the elderly interviewed	city___, county___, township___, village___, house number___
Neighbourhood/Village Committees Contact Person	
Neighbourhood/Village Committees contact number	
Investigator:	
Investigator's contact number:	
Interviewed Elderly No:	

The following information was completed by the elderly people interviewed:

**A. Basic information**		
A1	Your gender is?	① Male; ② Female
A2	How old are you?	_________
A3	Your registered residence is?	① Rural; ② Urban
A4	Your ethnicity is?	① Han; ② Minority
A5	The highest educational attainment you completed is?	① Elementary school and below; ② Junior high school; ③ Senior high school or technical secondary school; ④ Junior college; ⑤ Bachelor degree or above
A6	Your marital status is?	① Unmarried; ② Married; ③ Divorced; ④Widowed
**B. Health status**		
B1	Do you suffer from chronic diseases?	① Yes; ② No
B2	How do you perceive your current health status?	① Very good; ② Good; ③ Fair; ④ Poor; ⑤ Very poor
**C. Family and social security**		
C1	Your living arrangement is?	① Not alone; ② Alone
C2	The number of children you have raised is?	① 0; ② 1; ③ 2; ④ ≥3
C3	How is your relationship with your children?	① Very good; ② Good; ③ Fair; ④ Poor; ⑤ Very poor
C4	Your yearly income status	① ≤ 2000; ② 2001- 10000; ③ 10001- 30000; ④ 30001 -

		50000; ⑤ ＞ 50000
C5	Are you enrolled in medical insurance?	① Yes; ② No
C6	Are you enrolled in endowment insurance?	① Yes; ② No
**D. Accessibility of medical and health resources**		
D1	Walking time from current residence to the nearest healthcare center	① ＞ 15 minutes; ② ≤ 15 minutes; ③ Not have
D2	Satisfaction with the health service at the place of residence	① Very satisfied; ② Satisfied; ③ Fair; ④ Dissatisfied;⑤ Very dissatisfied
**E. The concept of elderly care**		
E1	Who should be relied on for elderly care?	① Oneself; ② Children; ③ Government
E2	Attitude towards living in a nursing home	① Support; ② Opposition; ③ Unclear
E3	Knowledge for the combination of medical and elderly care	① Never heard; ② Heard but not understood; ③ Have gained some understanding; ④ Know well
**F.** The willingness of the elderly to choose the elderly care mode		
F1	Which elder mode would you prefer?	① Home care; ② Community care; ③ Institutional care; ④ Mutual care; ⑤ Smart care

This is the end of your questionnaire. Thank you for your active participation and inputs. Wish you a happy life!

Appendix S2 The questionnaire revision process

Before the formal distribution of the questionnaire, a pre-survey was carried out with a group of one hundred retired residents who had been chosen with convenience sampling. Subsequently, appropriate revisions were made to the questionnaire based on the pre-survey results. Below are details of the questionnaire revision process:

Pre-revision	Post-revision	Reason for revision	Details of implementation
The questionnaire's "Family and Social Security" section did not contain a question on the relationship between the elderly and their children.	Question C3 was added to the section: ‘How is your relationship with your children?’ Options include: □ very good; □ good; □ fair; □ poor; □ very poor.	The pre-survey revealed that the relationship between elderly individuals and their children significantly impacts their willingness to select various elder care modes. The absence of comprehensive data in this area may obscure crucial factors that influence the choice of elder care modes."	Insert question C3 into the 'Family and Social Security' section of the questionnaire, and ensure that all investigators are fully aware of the significance of this newly added item.
The questionnaire did not make a clear distinction between older people's knowledge of the combination of medical and elderly care.	Question E3 has been added to the section on "Concepts of elderly care": How much do you know about the knowledge for the combination of medical and elderly care?The options were refined as: ① never heard; ② heard but not understood; ③ have gained some understanding; ④ know well.	The combination of medical and elderly care represents a unique mode of elderly care with distinct Chinese characteristics. Assessing older individuals' awareness of this model is essential for exploring their preferences and choices related to ageing.	Question E3 was added to the "The concept of elderly care" section and investigators were trained to ensure they understood and accurately interpreted this addition.
The contact details of the neighbourhood/village council were not asked for in the front part of the questionnaire.	A requirement for neighbourhood/village council contacts and contact details to be completed has been added to the opening section of the questionnaire.	Ensuring that reliable contact details are available in order to facilitate subsequent data verification or further contact is essential to improve data quality and the credibility of the study.	A special space was added at the beginning of the questionnaire for the contact person and contact number of the neighbourhood/village council.
